# Metabolomic Profiles of *Dinophysis acuminata* and *Dinophysis acuta* Using Non-Targeted High-Resolution Mass Spectrometry: Effect of Nutritional Status and Prey

**DOI:** 10.3390/md16050143

**Published:** 2018-04-26

**Authors:** María García-Portela, Beatriz Reguera, Manoella Sibat, Andreas Altenburger, Francisco Rodríguez, Philipp Hess

**Affiliations:** 1 IEO, Oceanographic Centre of Vigo, Subida a Radio Faro 50, Vigo 36390, Spain; beatriz.reguera@ieo.es (B.R.); francisco.rodriguez@ieo.es (F.R.); 2 IFREMER, Phycotoxins Laboratory, rue de l’Ile d’Yeu, BP 21105, F-44311 Nantes, France; manoella.sibat@ifremer.fr (M.S.); philipp.hess@ifremer.fr (P.H.); 3 Natural History Museum of Denmark, University of Copenhagen, Øster Voldgade 5-7, 1350 Copenhagen, Denmark; aaltenburger@snm.ku.dk

**Keywords:** *Dinophysis* physiology, lipophilic toxins, non-targeted analysis, metabolomics

## Abstract

Photosynthetic species of the genus *Dinophysis* are obligate mixotrophs with temporary plastids (kleptoplastids) that are acquired from the ciliate *Mesodinium rubrum*, which feeds on cryptophytes of the *Teleaulax-Plagioselmis-Geminigera* clade. A metabolomic study of the three-species food chain *Dinophysis-Mesodinium-Teleaulax* was carried out using mass spectrometric analysis of extracts of batch-cultured cells of each level of that food chain. The main goal was to compare the metabolomic expression of Galician strains of *Dinophysis acuminata* and *D. acuta* that were subjected to different feeding regimes (well-fed and prey-limited) and feeding on two *Mesodinium* (Spanish and Danish) strains. Both *Dinophysis* species were able to grow while feeding on both *Mesodinium* strains, although differences in growth rates were observed. Toxin and metabolomic profiles of the two *Dinophysis* species were significantly different, and also varied between different feeding regimes and different prey organisms. Furthermore, significantly different metabolomes were expressed by a strain of *D. acuminata* that was feeding on different strains of the ciliate *Mesodinium rubrum*. Both species-specific metabolites and those common to *D. acuminata* and *D. acuta* were tentatively identified by screening of METLIN and Marine Natural Products Dictionary databases. This first metabolomic study applied to *Dinophysis acuminata* and *D.acuta* in culture establishes a basis for the chemical inventory of these species.

## 1. Introduction

*Dinophysis* species produce diarrhetic shellfish poisoning (DSP) toxins and pectenotoxins (PTXs) and pose a major concern to public health and the aquaculture industry in Western Europe [1,2]. Their impact is particularly serious in the Galician Rías Baixas (NW Spain), site of intensive mussel (*Mytilus galloprovincialis*) production (up to 3 × 10^5^ metric tons per year; [3]). In this region, recurrent spring-summer proliferations of *D. acuminata*, followed in some years by those of *D. acuta*, cause lengthy harvesting bans whenever toxins in shellfish exceed regulatory levels [4]. Even though *Dinophysis* toxins (DTXs) emerged as a major risk since the 1970s, it was not until the recent cultivation of *D. acuminata* in the laboratory [5] that it became possible to undertake physiological studies of the growth and the toxin production dynamics of this organism. *Dinophysis* species are obligate mixotrophs that need light, nutrients, and live prey to survive and grow [6,7,8]. The feeding mechanism (earlier discovered by Hansen [9]) in *Phalacroma rotundatum* and *D. hastata* feeding on the ciliate *Tiarina fusus*) is a kind of phagocytosis (myzocytosis) where the prey content is sucked into the predator cell through a feeding peduncle. A similar structure is used by *D. acuminata* and *D. acuta* to feed on *M. rubrum* from which they temporarily retain its plastids (known as kleptoplastids) [10]. García-Cuetos et al. [11] argued that *Dinophysis* contained permanent plastids due to the different disposition of their thylakoids and the observation of a third membrane, in contrast with the four-membrane plastids of *M. rubrum*. Later publications however showed that these plastids are temporary and managed in different ways depending on the cryptophyte source [7,12,13]. Field studies have confirmed that *T. amphioxeia* and other *Teleaulax* species are the most common source of plastids in *Dinophysis* [14,15,16,17,18,19,20]. Nevertheless, the possibility of other kleptoplastid-bearing ciliate species (different from *Mesodinium*) acting as vectors of *Teleaulax*-like plastids to *Dinophysis* should not be excluded. This is supported by the finding of plastids from multiple algal sources in *Dinophysis* from Korean waters [17], and led other authors to hypothesize that different plastid-retaining oligotrichous ciliates, such as *Cyrtostrombidium*, *Laboea*, *Strombidium*, and *Tontonia* [21] could be alternative prey for *Dinophysis* [22]. *Mesodinium rubrum* is a phototrophic ciliate that feeds on cryptophytes [23] and retains their plastids and mitochondria [10,24]. The nature of this consortium has been a matter of debate over the last decade [25,26]. *Mesodinium rubrum* is able to feed on different cryptophyte genera, but higher growth rates in cultures are observed when species of the *Teleaulax/Plagioselmis/Geminigera* complex are added as prey [25,27]. Furthermore, whether there is a *Dinophysis*-specific selection or different growth response to different species (e.g., *M. major*), or even strains of *Mesodinium* remains an open question.

Recently, metabolomics has been introduced as a new approach to help in the understanding of metabolic characteristics, elucidating metabolic mechanisms and identifying metabolic biomarkers in a vast array of organisms [28]. Metabolome designates the array of metabolites, i.e., intra- and extracellular molecules resulting from enzymatic reactions, which a living organism is able to produce. Thus, a metabolome can be considered as a phenotypical expression, i.e., a chemical “snapshot” of an organism at a specific time, and is likely to change depending on external conditions [29,30]. Therefore, results that were observed in one single experiment should not be extrapolated to field conditions or other experiments, even when using the same species.

Studies on microalgae that are based on metabolomic analyses using mass spectrometry-based techniques are scarce and most of them have been carried out with diatoms [29]. Research on bioactive compounds in marine dinoflagellates has usually been related either to toxin production or pigments [31]. Metabolomics is a powerful screening tool to detect new compounds produced by these microalgae, including new toxins; to characterize them with their environment (environmental metabolomics, [32]); and, to describe their response to different stressors, such as climate change, and pollution [33]. It may be a suitable technique to identify differences and/or similarities between different strains of the same species [34], or to track species in different environments [35]. In the particular case of *Dinophysis* species, metabolomics may be helpful to understand similar and different behavioral traits between *D. acuta* and *D. acuminata*, the most relevant toxigenic species of *Dinophysis* in Western European coastal waters.

Some secondary metabolites of dinoflagellates, including lipophilic toxins, were first described as being produced by sponges. For instance, Tachibana et al. [36] isolated okadaic acid (OA) from the sponges *Halichondria okadai* and *H. melanodocia*, but suggested that OA could originate from a microorganism. This hypothesis was confirmed later by Murakami et al. [37], who identified OA production in the epibenthic dinoflagellate *Prorocentrum lima.* Antifungal extracellular metabolites, which inhibit the growth of *Aspergillus niger*, have been found in cultures of *Gambierdiscus toxicus* [38]. Anticancer compounds, such as a sulphated polysaccharide (GA3P), were isolated from *Gymnodinium* species [39,40], and amphidinolide H [41] and carbenolide [42] from *Amphidinium* sp.

The present study aimed to identify similarities and differences between the metabolomes of *D. acuta* and *D. acuminata* fed two strains of *M. rubrum* under different nutritional status (well-fed/prey limited) through non-targeted analysis with ultra-high-performance liquid chromatography (UHPLC) coupled to high resolution mass spectrometry (HRMS). Metabolites tentatively identified by screening of their mass against the Marine Natural Products Dictionary [43] or METLIN [44] were associated with specific culture conditions, and could be used as biochemical markers of both physiological conditions and species-specific responses to transient environmental conditions. This is the first time that metabolomic techniques were applied to study *Dinophysis*.

## 2. Results

### 2.1. Phylogenetic Analysis

**Figure 1:**
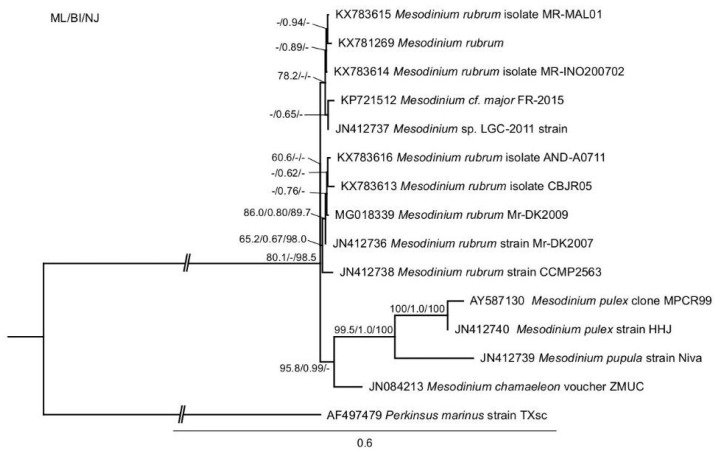
Phylogeny of *Mesodinium* inferred by maximum likelihood analysis of SSU-rDNA sequences. Numbers at nodes represent the bootstrap values of maximum likelihood out of 1000 replicates; the posterior probability of Bayesian analysis; and, the bootstrap values of neighbor-joining out of 10,000 replicates. Not resolved branches are marked with minus (−). The scale bar corresponds to six substitutions per 100 nucleotide positions. The branch to the out-group is not to scale.

The phylogenetic analysis of *M. rubrum* was consistent with previous findings and showed four distinct clades that were represented by the *Mesodinium rubrum* complex, *M. chamaeleon*, *M. pulex*, and *M. pupula* [28,45]. The newly sequenced Danish (MrDK-2009) SSU-rDNA showed a 99.7% pairwise sequence identity with that of the Spanish (AND-A0711) strain (Figure 1).

### 2.2. Growth Curves

**Figure 2:**
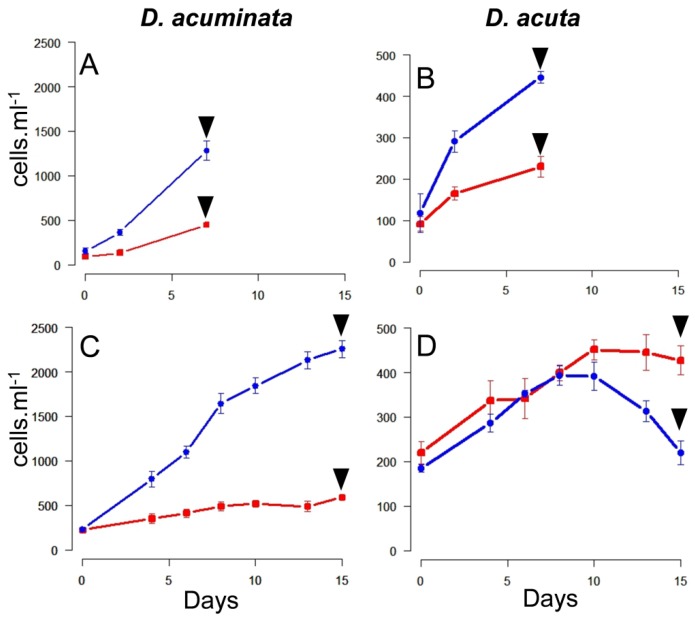
Growth curves during the first (**A**,**B**) and second (**C**,**D**) phase of Experiment 2. *Dinophysis acuminata* (**A**,**C**); *D. acuta* (**B**,**D**). Arrows indicate the harvesting day, blue and red lines correspond to *Dinophysis* cells fed Spanish and Danish *Mesodinium rubrum*, respectively. (Notice the different scale in the *Y*-axis for both species).

During the first phase of Experiment 2, all the triplicate flasks of well-fed *Dinophysis* still contained *Mesodinium* cells after seven days. Maximal specific growth rates (µ_0–7_) and yield were found in cultures of *D. acuminata* fed Danish (0.42 day^−1^; 1.2 × 10^3^ cells L^−1^) and Spanish (0.32 day^−1^; 500 cells L^−1^) *Mesodinium*. Lower values were obtained in the case of *D. acuta* fed either Danish (0.27 d^−1^; 500 cells L^−1^) or Spanish (0.18 day^−1^; 200 cells L^−1^) *Mesodinium* (Figure 2A,B).

During the second phase of Experiment 2, ciliates were exhausted by day 7 in all the triplicate flasks, except those of *D. acuta* fed Danish *M. rubrum* (Figure 2C,D). Results on specific growth rates and yields showed maximal values for *D. acuminata* fed Danish *M. rubrum* (µ_0–8_ = 0.25 day^−1^). Nevertheless, *D*. *acuminata* fed Spanish *M. rubrum* (µ_0–10_ = 0.08 day^−1^) and *D. acuta* fed Danish *M. rubrum* (µ_4–8_ = 0.08 day^−1^) had a very poor growth and *D. acuta* fed Spanish *M. rubrum* had hardly any growth (µ_6–10_ = 0.07 day^−1^). Thus, *D. acuta* did not seem to feed well on Danish ciliate prey and culture densities declined from day 10 onwards. The opposite was observed in *D. acuminata*, which reached a density of ~2200 cells mL^−1^ by the end of Experiment 2 (Figure 2C).

The biomass of *D. acuta* and Danish *M. rubrum* harvested for toxins and other analyses on the second phase of Experiment 2 represented 81.6 ± 4.0% and 18.4 ± 4.0% of the total pellet biomass, respectively. In contrast, during the first phase of Experiment 2 these percentages were 93.5 ± 1.4% and 6.5 ± 1.4% for *D. acuta* and Danish *M. rubrum*, respectively, *D. acuta* being well fed in both situations (Table 1).

### 2.3. Quantitative Toxin Analysis Using Low Resolution Mass Spectrometry

*Dinophysis acuminata* and *D. acuta* showed different toxin profiles. In both experiments, *D. acuta* produced OA, DTX2, and PTX2, whereas *D. acuminata* produced only OA (Table 2). Supernatants were not analyzed in this study and PTX2 derivatives could not be studied due to toxin degradation during the hydrolysis procedure [46]. Toxin quantification, pre- and post-hydrolysis, was used to indicate the potential presence of esters of the OA-group toxins in the samples from the two experiments.

During mid-exponential phase in Experiment 1, hydrolyzed samples of *D. acuminata* had 1.6 times more OA per cell than the non-hydrolyzed ones. *Dinophysis acuminata* contained 2.4 times more free OA (22 pg cell^−1^) and three-fold more total OA (35 pg cell^−1^) than *D. acuta*, the latter, however, contained a significant cell quota of PTX2. Results of a one-way analysis of variance (ANOVA) when comparing both triplicates were statistically significant (F(9.96) > 7.71; *p* < 0.05). In the case of *D. acuta*, total OA was 33% higher than free OA, whereas the amounts of DTX2 were nearly the same.

Estimated biovolumes of the different species were 65 µm^3^ for *Teleaulax amphioxeia*, 2169 µm^3^ and 2900 µm^3^ for the Spanish and Danish *Mesodinium*, respectively, and 16,752 µm^3^ and 54,333 µm^3^ for *D. acuminata* and *D. acuta* respectively. Thus, the Danish *Mesodinium* was approximately 33% larger than the Spanish one, and *D. acuta* approximately three times larger than *D. acuminata.*

During the first phase of Experiment 2, *D. acuta* contained more OA per cell than *D. acuminata*. During the second phase of the experiment, cells from prey-limited cultures of *D. acuta* had more OA and DTX2 per cell than the well-fed cells and the same pattern was found for OA in *D. acuminata* cultures. Toxin per cell values under both nutritional conditions was compared.

ANOVA results were significant for OA in *D. acuta* fed Spanish *M. rubrum* (F(20.99)>10.13; *p* < 0.05) and *D. acuminata* fed both strains of *M. rubrum*, (F (11.02)>10.13; *p* < 0.05) with the Spanish and (F (16.70)>7.71; and, *p* < 0.05)with the Danish *M. rubrum*. For DTX2 and PTX2, ANOVA results were significant only in the case of *D. acuta* fed Spanish *M. rubrum*, these results being (F (12.91) > 10.13; *p* < 0.05) for DTX2 and (F (13.38)>10.13; *p* < 0.05) for PTX2. Total OA content per cell in prey-limited *D. acuminata* cultures (close to stationary phase) were on average 3.6 (Danish *Mesodinium*) and 3.5 times (Spanish *Mesodinium*) higher than well-fed cells (closer to exponential growth phase). In fact, ANOVA results were only significant for OA in these two cases, being (F (55.53)>10.13; *p* < 0.05) and (F (59.59)>7.71; *p* < 0.05) for the Spanish and Danish *M. rubrum* feeding, respectively. In the case of total and free DTX2 in *D. acuta*, again results were significant only with *D. acuta* fed Spanish *M. rubrum* (F (17.13)>10.13; *p* < 0.05). When comparing hydrolyzed and non-hydrolyzed samples, ANOVA analysis was significant only for *D. acuta* well-fed with Spanish *M. rubrum*.

#### Esters of OA-Group Toxins

The quantitative analysis using low resolution mass spectrometry suggested a large difference between total and free OA in *D. acuta* well-fed with Spanish *Mesodinium*. Indeed, spectra that were acquired from targeted high-resolution tandem mass spectrometry in both positive and negative ionization modes strongly suggested the presence of an OA ester, which had not been previously described in *D. acuta* [47].

### 2.4. Data Treatment for Non-Targeted Liquid Chromatography High Resolution Mass Spectrometry

**Figure 3:**
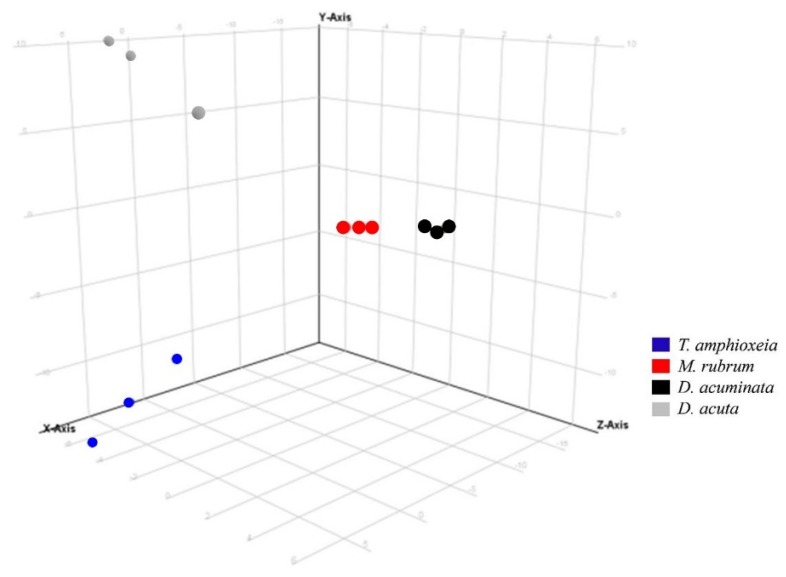
Principal component analysis of features detected in negative ionization mode according to the variable “species” in Experiment 1. First, second and third principal components (PC1, PC2, and PC3, the three components representing the largest fraction of the overall variability) are displayed on the *X*, *Y*, and *Z*-axis. Variability explained by each component: PC1 (*X*-axis) 57.1%, PC2 (*Y*-axis) 26.5%, PC3 (*Z*-axis) 11.1% (total 94.7%). *Nota bene*: biological replicates of each species group together, indicating the consistent differences in chemical profiles of each species.

Three variables were considered in the data analysis: (i) “species” (*D. acuta*, *D. acuminata*, *M. rubrum* and *T. amphioxeia*); (ii) “prey origin” (Danish and Spanish *M. rubrum* strain) and (iii) “nutritional status” (well-fed and prey-limited). Variable “species” was used in Experiment 1 to separate the response of different organisms in the principal component analysis (PCA): The *X*-axis explained the highest variability, i.e., 45.66% in ESI^+^ mode (data not shown) and 57.08% in ESI^−^ mode (Figure 3).

**Figure 4:**
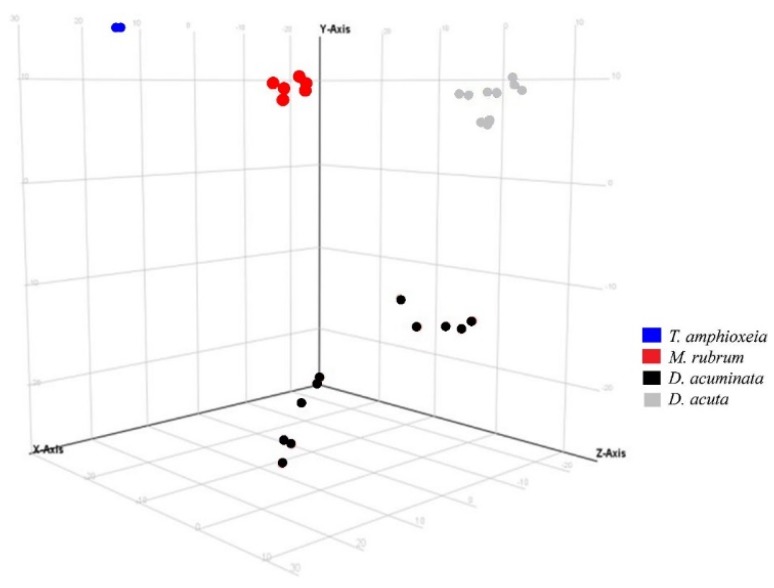
Principal component analysis of features detected in negative ionization mode according to the variable “species” in Experiment 2. First, second and third principal components (PC1, PC2, and PC3, the three components representing the largest fraction of the overall variability) are displayed on the *X*, *Y*, and *Z*-axis. Variability explained by each component: PC1 (*X*-axis) 45.4%, PC2 (*Y*-axis) 21.9%, PC3 (*Z*-axis) 15.0% (total 82.3%). *Nota bene*: biological replicates of each species group together, albeit somewhat less than in Experiment 1 (Figure 3) due to the increased complexity of Experiment 2 (additional Danish *Mesodinium* and different nutritional status).

A similar response was observed using the same variable in Experiment 2 (Figure 4). Species separation was clearly visible, with replicates of *Mesodinium*, *Teleaulax*, and the two species of *Dinophysis* forming separate clusters in principal component analysis. Intraspecific differences in each of the two *Dinophysis* species were larger than in Experiment 1, most likely due to the increased complexity of Experiment 2 since this one included treatments with different prey origins and nutritional status.

**Figure 5:**
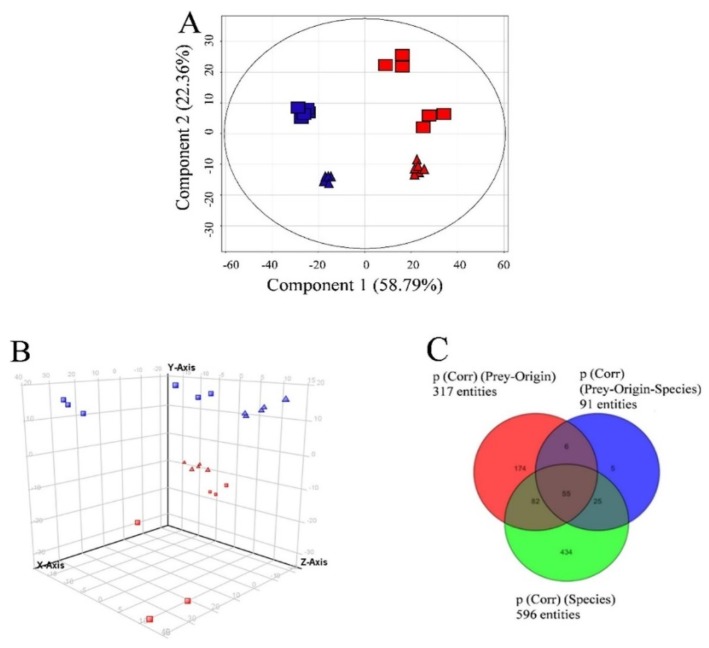
(**A**) Principal component analysis of features detected in the two *Dinophysis* species (excluding prey species) in negative ionization mode showing variables “species” and “prey origin” in Experiment 2. First and second principal components (the two components representing the largest fraction of the overall variability) are displayed on the *X* and *Y*-axis. Variability explained by each component: component 1 (*X*-axis 58.8%), component 2 (*Y*-axis) 22.4% (total 81.2%). *Dinophysis acuminata* and *D. acuta* (“species”) are represented in red and blue color, respectively and the origin of *Mesodinium* used as prey are represented as triangles and rectangles for Denmark and Spain, respectively; (**B**) Principal component analysis of features detected in positive ionization mode in Experiment 2 (grouping by species and prey origin). First, second and third principal components (PC1, PC2, and PC3, the three components representing the largest fraction of the overall variability) are displayed on the *X*, *Y*, and *Z*-axis Variability explained by each component: PC1 (*X*-axis) 37.7%, PC2 (*Y*-axis) 26.7%, PC3 (*Z*-axis) 9.9% (total 74.3%). *Nota bene*: *D. acuta* samples in top half of the graph and *D. acuminata* samples in the bottom half of the graph, i.e., separation by species driven by the second component (*Y*-axis). “Species” and “prey origin” are represented in the figure as for A; (**C**) Venn diagram explaining overlap of features specific to species, prey-origin, and interactions of these parameters (see also Supplementary Table S4A–C).

When *Teleaulax* and *Mesodinium* were excluded from the dataset, *D. acuminata* and *D. acuta* were clearly separated by the variable “species” (Figure 5A), also showing the highest number of species-specific compounds (*p* < 0.001; see Tables S3A–C and S4A–C for compounds specific to both *Dinophysis* species ranked by increasing *p-*values). This example also highlights the meaning of each principal component, as component 1 (Figure 5A) corresponds to the separation according to species, whereas component 2 corresponds to grouping according to prey origin. As component 2 explains less than half of the variability as compared to component 1, the variable “species” can be considered most important, followed by prey origin.

**Figure 6:**
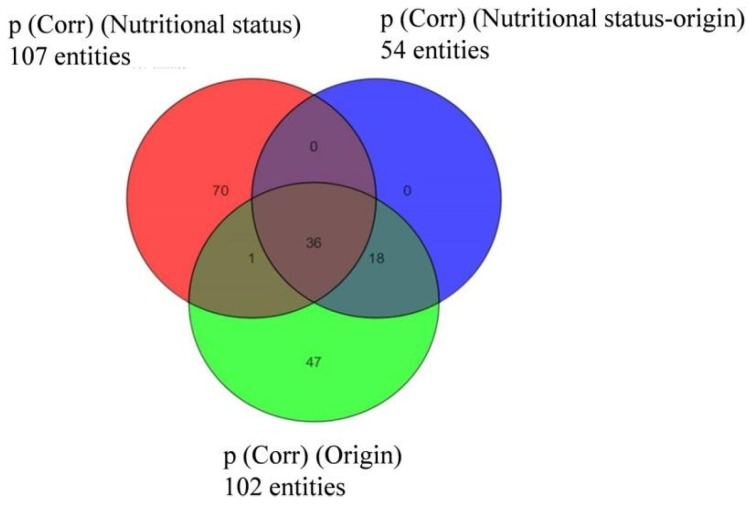
Venn diagram indicating the distribution of compounds specific to or common within groups of *Dinophysis acuminata* depending on “prey origin” and “nutritional status” from Experiment 2 (ESI^−^).

In the case of *D. acuminata*, many compounds (107) were specific to one of the two nutritional conditions (Figure 6). Still, taking the previous observations into account, only metabolites appearing under “species” and “prey origin” variables were considered in the following sections.

While the above analyses did not investigate the nature of the features that were detected in the non-targeted HRMS analysis, the following sections give an overview of the tentative identifications after comparisons with the databases METLIN and/or Marine Natural Products Dictionary.

#### 2.4.1. Compounds with an Already Known Physiological Meaning

##### Common to All Species

*Dinophysis* cells that were harvested from Experiment 1 had been previously washed to eliminate the remains of cryptophytes and *M. rubrum* from the cultures, but this step was not repeated for Experiment 2. For this reason, only compounds appearing in both experiments are shown in Table S1A,B.

Within the compounds common to all species, the one with the highest score was a molecule (Compound ID: S1A-3) which showed the maximal relative abundance in *T. amphioxeia*, decreasing progressively from *M. rubrum* to *Dinophysis*. When considering the normalized abundance of this compound in both experiments, its transfer through the food chain from *T. amphioxeia* to *Dinophysis* is plausible. This compound was previously found in the marine dinoflagellate *Heterocapsa circularisquama* and at a concentration ≥0.5 µg mL^−1^ it showed cytolytic activity toward oyster heart cells [48]. Another compound common to all species in both experiments was a galacto-glycerolipid-like compound (Compound ID: S1A-2 and S1B-8), in particular, a mono-galactosyl-diacyl-glycerol (MGDG 18:5), which had large quantities of polyunsaturated fatty acids. Mono- and di-galactosyl-diacylglycerols (MGDGs/DGDGs) are present in cyanobacteria and chloroplasts, and are used to preserve the Photosystem II (PSII) components. In higher plants, changes in the mono- and di-galactosyl-diacylglycerol ratios (MGDG/DGDG) are related to environmental changes that are affecting the structure of thylakoid membranes [49]. In fact, MGDGs with different structures (MGDG 20:5/16:3) appeared in Experiment 2 (Compounds ID: S1B-6, S1B-10, and S1B-11). All three compounds had the highest content in *T. amphioxeia*, and this relative content decreased from *Mesodinium* to *Dinophysis* (particularly in the case of *D. acuta*). Different structures of these lipids (MGDG and DGDG) have been suggested to be responses to changes in temperature [50]. Such responses were observed in different strains of *Pyrocystis lunula*, *P. noctiluca*, and *P. fusiformis*, in which DGDG (20:5/18:4) and DGDG (20:5/18:5) were the dominant galactolipids in all the strains at temperatures of 25 °C and 15 °C, respectively, whereas MGMG (20:5/18:5) was predominant at 15 °C.

In our study, the presence of glycerolipids was generally reduced under prey-limited conditions, but these lipids did not seem to be specific to either *D. acuminata* or *D. acuta*.

Compound ID: S1B-4, which was a minor compound in both species of *Dinophysis* fed Danish *M. rubrum* (also present in all the species), was identified as a phosphatidyl glycerol (PG)-like molecule (through the METLIN database albeit with a low score of 53.04%)*.* This phospholipid is an important component of thylakoid membranes [51], and its absence produces photosynthesis photo-inhibition [52]. After the description of the two previous compounds, it is worth mentioning that from algae to higher plants, P-limitation leads to an increased galactoglycerolipid/phosphoglycerolipid ratio [53,54]. However, the same compound was also identified as the methylester of melanodocin, an isomer of acanthifolicin (Marine Natural Products Dictionary, score of 99.3%). Acanthifolicin is a sulphide of okadaic acid, and this compound had not been reported from dinoflagellates but had been initially isolated from the sponge *Halichondria melanodocia*. However, the presence of this compound in all species, including *Teleaulax amphioxeia*, supports the hypothesis of a (PG)-like molecule. In any case, further studies are necessary to confirm the compound identity and gain new insights into its role.

##### Compounds Exclusive to the Ciliate *Mesodinium*

No compounds that were specific to the *M. rubrum* strains were found. This result may well be related to some residual contamination from *T. amphioxeia* in ciliate cultures, but also to the fact that *Mesodinium* keeps the full set of cryptophyte organelles (nucleus, mitochondria, chloroplast) and the cryptophyte transcriptional machinery active after sequestration [26,55].

##### Compounds Exclusive to *Teleaulax amphioxeia*

Two compounds that were specific to the cryptophyte *T. amphioxeia* (Table S2, compounds ID: S2-1 and S2-2) were found. One of them (Compound ID: S2-2) matched the group of phospholipids, which form part of eukaryotic cell membranes with the suggested function of maintaining their fluidity [56].

##### Compounds Exclusive to One of the Two Species of *Dinophysis*

A glycerol-like compound (Compound ID: S7A-1) was identified only in *D. acuminata* from Experiment 2 (Table S7A). This compound is required for the synthesis of triacylglycerols, phospholipids, and glycolipids. Amongst glycolipids, glycosphingolipids act as cell adhesion mediators and signal transduction modulators. When they are associated with other proteins (glycosynapse), they act as functional groups through which glycosylation-dependent cell adhesion coupled with signal transduction takes place [57]. These glycosphingolipids, together with other polysaccharides, are involved in membrane formation and were found to be overexpressed during bloom conditions in field populations of *Levanderina fissa* [58]. Stigmastane-like compounds (Compounds ID: S10-3 and S10-4) were only present in well-fed cells of *D. acuminata* (fed Danish *M. rubrum*; Table S10). The cellular content of this kind of sterols was found to increase in *Gymnodinium* sp. cultures when they reached the stationary phase [59]. Maximum levels of other sterol lipids, e.g., compounds ID: S11B-1 and S11B-4, were found in starved cells of *D. acuminata* (Danish *M. rubrum*) (Table S11B).

Protochlorophyllide (Compound ID: S11B-9), which is an ester-like molecule that is involved in the biochemical route for the synthesis of chlorophyll compounds [60], was only found in prey-limited cells of *D. acuminata* initially fed Danish *M. rubrum* (Table S11B), together with a phaeophytin a-like compound (Compound ID: S11B-5; chlorophyll a degradation product).

#### 2.4.2. Compounds First Found in Other Marine/Terrestrial Organisms

Many of the compounds that were found in either or both *Dinophysis* species were tentatively identified as polyketides. Polyketides are a structurally diverse group that is constituted by a carbon chain synthesized by a family of enzymes called polyketide synthases (PKSs). Genes coding for PKSswere first cloned and sequenced in bacteria and fungi, and later identified and associated with the dinoflagellate *Karenia brevis* by Snyder et al. [61,62].

##### Compounds Present in Both Species

Several compounds that were identified in both species of *Dinophysis* from Experiment 2 (Table S5A,B) were tentatively identified as having first been found in marine invertebrates (corals and sponges). For example, the lipophilic toxin OA (Compound ID: S5A-1) was first isolated from the sponges *Halichondria okadai* and *H. melanodocia* [36]; 5,8-epidioxysterols (Compound ID: S5A-3) were isolated from corals (*Sinularia flexibilis*, [63]), which may feed on toxigenic dinoflagellates [64].

##### Compounds Exclusive to One of the Two Species of *Dinophysis*

*Dinophysis acuminata*

Several compounds previously isolated from sponges were tentatively identified in *D. acuminata* (Table S7B, Compound ID: S7B-1), but were not related with a particular “prey-origin” nor “nutritional-status” condition. Other compounds that were first isolated also in sponges and skates (genus *Raja*) were identified in well-fed cells of this species (Compound ID: S10-5), regardless of the *Mesodinium* strain that was used as prey (Table S10). Different alkaloid-like compounds previously found in starfish and sponges were found in food-limited *D. acuminata* cells (Table S11A,B, compound ID: S11B-11).

*Dinophysis acuta*

Brevetoxins and OA-related toxins may be classed as polyketides [65]. While OA was present in both *D. acuta* and *D. acuminata*, a peak of an isomer appeared just a few seconds after OA in *D. acuta* only, which corresponded to dinophysistoxin-2 (DTX2). Dinophysistoxin 1 (DTX1) and DTX2 often co-occur with OA, but the two DTXs rarely co-occur. This is probably due to the fact that DTX2 has a stereochemistry opposite to that of DTX1 at their C-35 [66], which suggests a significant difference in the biosynthetic pathway. 

In agreement with results from targeted analysis by low resolution mass spectrometry (Table 2), PTX2 was also specific to *D. acuta* in the non-targeted analysis (Table S6B, compound ID: S6B-5). The abundance of PTX2 was slightly higher in prey-limited than in well-fed cells, in particular, for the cells that were fed Spanish *M. rubrum*.

A compound (Compound ID: S9A-4) that was tentatively identified as Prorocentin was the most abundant of all the compounds specific to prey-limited *D. acuta* fed Danish *M. rubrum*. Prorocentin, which was first isolated from the toxic dinoflagellate *Prorocentrum lima* [67], is a polyketide suggested to share a biosynthethic pathway with OA.

Putative compounds that are found only in *D. acuta* (i.e., Compound IDs: S6B-2 and S6B-9) were described in the sponge *Sigmosceptrella* [68], and were later found in other marine organisms, such as the coral *Klyxum flaccidum* ([69], Table S6A,B).

A compound originally described in sharks (Compound ID: S8-1) was found in well-fed (Danish *M. rubrum*) cells of *D. acuta* (Table S8). However, when food was limiting, a putative anti-feedant compound (Compound ID: S9A-1) that was initially isolated from the cyanobacteria *Lyngbya majuscula* [70], was identified together with other compounds firstly identified in sponges, molluscs, and bacteria (Table S9A,B).

## 3. Discussion

### 3.1. Growth Curves

Growth rates that were obtained in Experiment 2 for both *Dinophysis* species demonstrated the significant effect of prey source (Spanish or Danish *M. rubrum* strain). Interestingly, *D. acuminata* showed the highest growth rates under both nutritional conditions when it was initially fed Danish *M. rubrum*. Only 0.03% of genetic dissimilarity was observed when comparing both *Mesodinium* strains, but the biovolume of the Danish strain was 33% larger than that of the Spanish one. In this regard, it is well known that prey size and morphological features affect the selectivity of predators towards specific prey organisms. This phenomenon has previously been demonstrated for the heterotrophic dinoflagellates *Oxyrrhis marina*, *Protoperidinium pallidum*, and *P. steinii:* when they were fed algae with different sizes, their growth was mainly supported by the larger-sized specimens [71,72].

### 3.2. Toxin Quota and Target Analysis

Toxin content per cell of all toxins was higher in *Dinophysis* cultures during the prey-limited phase (Experiment 2), except for PTX2 in *D. acuta* fed Danish ciliate, which was probably related to the poor physiological state of that culture (Table 2). Earlier experiments with *D. acuminata* [73] with *D. fortii* [74] showed that prey limitation reduced its growth and increased toxin content per cell due to an imbalance between cell division and toxin production rates. The different results that were observed for *D. acuta* are in accordance with the observations on its feeding behavior: *D. acuta* did not feed well on the Danish ciliate, a fact that would also explain the lack of differences between well-fed and prey limited phases (Table 2, Experiment 2). In contrast, *D. acuta* reached the highest content of OA per cell when feeding on Spanish *Mesodinium* (Table 2, Experiment 2).

Toxin content per cell observed in both *Dinophysis* species during Experiments 1 and 2 (*D. acuta:* 70 pg PTX2 and 75 pg OA cell^−1^; *D. acuminata*: 32 pg OA cell^−1^ in hydrolyzed samples) was significantly higher than any ever-recorded in picked cells of *D. acuminata* and *D. acuta* strains from the Galician Rías. Earlier studies on *Dinophysis* strains from this region showed maximum values of 9.4, 6.6 and 6.1 pg cell^−1^ of OA, DTX2, and PTX2, respectively [75], and a simpler profile with only PTX2 (32.3 pg PTX2 cell^−1^) in *D. acuta* from another year [76].

Toxin analysis that was carried out during the baseline experiment (Experiment 1) showed the same toxins as in Experiment 2, but their proportions were somewhat different and the cell-toxin quota was lower in Experiment 1. Thus, PTX2 and total DTX2 toxin content in *D. acuta* was only 22 and 4 pg cell^−1^, respectively, in Experiment 1, while the largest amount of these toxins were 70 and 30 pg cell^−1^, respectively, during parts 1 and 2 of Experiment 2. During Experiment 1, OA in *D. acuminata* reached the highest content per cell (~21.5 pg OA cell^−1^), similar to those that were acquired during Experiment 2. Contrarily, inExperiment 2, it was *D. acuta* that reached the highest content of OA per cell (~76 pg OA cell^−1^), while during Experiment 1 OA toxin content per cell was found in a minor quantity (~9 pg OA cell^−1^). The time lag between Experiments 1 and 2 was approximately three months, so it is unlikely that toxin loss occurred in *D. acuminata* cultures, as has been observed with other dinoflagellate cultures. As an example, the losses of toxin content in a strain of *Alexandrium lusitanicum* (=*A. minutum*) have been related to slower growth in the laboratory [77].

### 3.3. Semi-Quantitative Non-Targeted Analysis: Comparison of Larger Parts of Metabolomes

A multivariate analysis was used to estimate differences between phenotypes, at the chemical level, in relation to three variables: “species”, “prey origin”, and “nutritional status”. The variable “species” led to the highest separation within samples, followed by “prey origin”. The “nutritional status” did not provide clear-cut results in the case of *D. acuta*, neither during the growth experiments nor in the PCA analysis. In contrast, in the case of *D. acuminata*, distinct results were obtained depending on the “nutritional status”. Statistical analysis that was carried out here gave enough separation between the organisms, however future studies should include partial least square–discriminant (PLS-DA) analysis and Variable Importance in Projection VIP score plot, in addition to PCA to underscore such separation.

Only a few lipid-derivatives could be tentatively identified in every species in the present study and were likely transferred through the three-species food chain (*Teleaulax-Mesodinium-Dinophysis*). Metabolomic studies aim to unveil a comprehensive view of the metabolome, i.e., the set of metabolites a biological entity may produce, although datasets in this field can be convoluted [78]. This part of the study would fit in the field of *partial ecometabolomic studies (PEM)*, a term coined by Sardans et al. [79], which encompasses ecophysiological metabolomic studies focused on the identification of metabolites that are produced in response to specific biotic/abiotic effects on organisms. Formally speaking about metabolomics, intra- and extracellular metabolites should be analyzed [80]. The main objective of this work was to identify intracellular compounds (whether by formulae or by name) that were (i) common to all the species that were used in this study (*T. amphioxeia*, *M. rubrum*, *Dinophysis* species); (ii) specific to some of them; or, (iii) species-specific. It was not intended to be a comparison between both batches of samples (Experiments 1 and 2) as these types of analysis are “snapshots” of the cell at the time of being harvested, and they were, indeed, harvested at different times. Due to the diversity of the metabolome, it is not possible to identify all metabolite types [33]. This study represents a first application of these next-generation technologies to *Dinophysis*, and should be useful, among others, to identify molecular markers that are involved in predator-prey recognition and/or *Dinophysis* identification.

Regarding biological results, maximum yields were obtained in the *D. acuminata* strain fed Danish *M. rubrum*. Contrary effects were seen when the population of *D. acuta*, feeding on the same prey, declined during the second part of Experiment 2. Therefore, important questions regarding Experiment 2 were: could such compounds be involved in predator-prey recognition? If so, do these compounds belong to the Danish *M. rubrum* or are specific for *D. acuminata* or *D. acuta*?

Different alkaloids and lipids were mainly identified in prey-limited cells of *D. acuminata* that were initially fed Danish *M. rubrum*. In the case of *D. acuta*, the putative characterization of a compound with anti-feeding properties (Compound ID: S9A-1) could be associated with the shift to early stationary and senescent phases in this species. In fact, the release of secondary metabolites by dinoflagellates, and their effects have gained much attention during the last years. The term “allelopathy” can be understood as an adaptation [81] related to the secretion of secondary metabolites and other biochemicals [82]. Particularly, the lipopeptide Malyngamide A was shown to have anti-feeding properties that deterred the uptake by two species of coral red fishes of artificial food (mixture of water, agar, and powdered green alga) containing the lipopeptide [83]. Many identified features were first isolated in higher marine organisms implying roles in various ecological relationships. Okadaic acid was first isolated from the sponges *Halichondria okadai* and *H. melanodocia* [36], so it could well be that compounds found in other marine organisms in fact were derived from smaller-sized cells. Toxins from certain strains of the dinoflagellate *Karlodinium veneficum* displayed anti-feeding properties against the copepod *Acantia atonsa* [84]. In this context, Denticulatin A, which has been tentatively identified in both *Dinophysis* species (Compound ID: S4A-38), can be mentioned as a possible ichtyotoxic agent due to its polypropionate character.

Regarding the issue of how *Dinophysis* can recognize the ciliate *M. rubrum* as a prey, different glycolipids (e.g., Glycerol 1, 2-dialkanoates in *D. acuminata*) and other compounds identified only tentatively by their formulae were found. Raho et al. [13] used different lectin markers to detect the presence of cell-surface binding carbohydrates in Galician strains of *D. acuminata* and *D. acuta*. Such lectin markers showed positive results in both *Dinophysis* as well as in *M. rubrum* cells, suggesting that lectins could play a key role in predator-prey recognition. Wood-Charlson et al. [85] showed that the cell surface of *Symbiodinium* contained glycan ligands (α-Mannose/α-Glucose and α-Galactose residues), which bound with two different lectins (ConA and Jac) that were found in *Fungia scutaria* coral larvae, meaning that some recognition mechanism is playing an important role in the coral/dinoflagellate symbiosis. Several recent studies pointed out the excretion by *Dinophysis* of a mucus trap to catch its prey [86,87,88]. Mafra et al. [86] demonstrated that the “toxic substance” in the mucus was not a lipophilic toxin. These authors suggested that excreted toxic substances, together with the mucus, were part of *Dinophysis* feeding strategies; however, prey capture by *Dinophysis* using a mucus trap was not observed in the present study. Finally, we repeat that all the identifications in this study must be considered as tentative, since no reference compounds are available for many natural products, and full scan high resolution mass spectrometry only allows for an initial comparison with existing databases, but without complete structural confirmation. Further studies need to be carried out to follow up on these results and confirm the actual identity of the metabolites.

## 4. Material and Methods

### 4.1. Cultures

**Figure 7:**
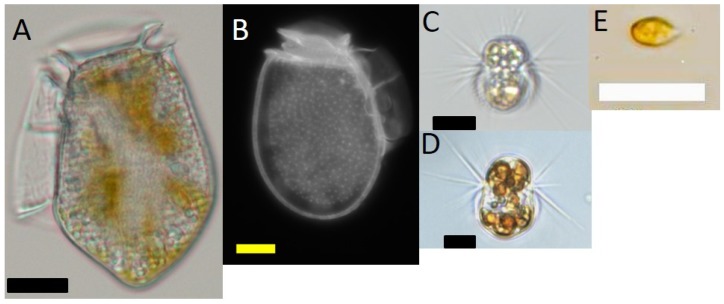
Light microscope micrographs of (**A**) *Dinophysis acuta;*(**B**) *D. acuminata*; (**C**) *Mesodinium rubrum* from Spain; (**D**) *M. rubrum* from Denmark; (**E**) *Teleaulax amphioxeia*. Scale bars: (**A**) (20 μm); and, (**B**–**E**) (10 μm).

*Dinophysis acuminata* (strain VGO1349) was isolated from Ría de Vigo in July 2016 and *Dinophysis acuta* (VGO1065) from Ría de Pontevedra in October 2010. A strain of the ciliate *Mesodinium rubrum* (AND-A0711, Acc. Number KP142651) and the cryptophyte *Teleaulax amphioxeia* (AND-A0710; Acc. Number KP142646) were isolated from Huelva, Southwest Spain, in 2007. Cultures of *M. rubrum*, fed the cryptophyte *T. amphioxeia*, were periodically given to *Dinophysis* as prey. An additional strain of *M. rubrum* (MrDK-2009, Acc. Number MG018339), which was isolated from Helsingør Harbor (Denmark) during summer 2009, was used in this study. Images of these organisms are shown in Figure 7.

### 4.2. DNA Extraction, Polymerase Chain Reaction (PCR), 18S Sequencing and Phylogenetic Analysis of Mesodinium

A culture of *M. rubrum* (Mr-DK2009) fed the cryptophyte *Teleaulax acuta* was starved for two weeks until no prey could be detected. Single cells were picked and transferred to 0.2 mL PCR tubes containing 100 µL of milli-Q water and 10% (*w*/*v*) Chelex 100 (Sigma-Aldrich #C7901, St. Louis, MO, USA). For DNA extraction, the PCR tubes were vortexed for 5 s, spun down in a microcentrifuge for 10 s, and subsequently incubated at 95 °C for 20 min [89]. After incubation, the tubes were centrifuged again for 10 s and stored at 4 °C until use for PCR reactions.

DNA extract (2 µL) was used as template in the subsequent PCR reactions. The following primer pairs were used: 4617F-Meso580R; Meso245-UNIDEUK1416R; Meso580F-Meso1480R; Meso1200F-Meso28S_R; and, ITS1-Dir-2CR (see Table 3). PCR reactions were carried out in 25 µL reaction-tubes containing, 1.5 mM MgCl_2_, 0.8 mM dNTPs (VWR #733-1363), 0.5 units polymerase (VWR #733-1301), 0.4 µM primers using the following reaction settings: 2 min at 95 °C, followed by 40 cycles: 95 °C for 30 s; 57 °C for 30 s; 72 °C for 50 s; and finally, 5 min at 72 °C.

PCR products were tested on a 2% agarose gel and purified with a QIAquick PCR purification kit (Qiagen #28106). Purified samples were sent to Macrogen (Macrogen Europe, Amsterdam, The Netherlands) for Sanger sequencing in both directions. Sequence analysis (trimming, assembly, BLAST) was done with Geneious version 10.1.3. For the phylogenetic analysis, additional sequences of the SSU rRNA gene were downloaded from GenBank and were aligned using T-Coffee, as implemented by intcoffe.crg.cat. The alignment included 2780 characters and was uploaded to the South of France bioinformatics platform for PhyML 3.0 analysis with Smart Model Selection (best model was GTR + G + I), using the Akaike Information Criterion and performing 1000 bootstrap replicates [90,91]. Bayesian Inference was performed with MrBayes 3.2.6 using a GTR + I + Γ model, as implemented in Geneious^®^ 10.2.2 (Biomatters Ltd., Auckland, New Zealand) [92]. The following settings were used: four simultaneous Markov chain Monte Carlo (MCMC) run for 1,000,000 generations, sampling every 100 generations. The first 25% of trees were discarded as burn in. Finally, a neighbor-joining tree was build, using the Jukes-Cantor genetic distance model and 10,000 bootstrap replicates, as implemented in Geneious^®^ 10.2.2.

### 4.3. Biovolume

Measurements of the organisms that were studied (cryptophytes, *Mesodinium*, and *Dinophysis*) were carried out to estimate their biovolumes. These estimates were used to normalize data related to the identified features (for “feature” meaning explanation see below at Section 4.7.1). Imaging of 30 specimens of each *Dinophysis* species was carried out with an Axiocam HRC digital camera (Zeiss, Oberkochen, Germany) and measurements that were taken under a light microscope (Leica DMR, Germany) at 630 X magnification. *Dinophysis acuta* geometric shape was considered equivalent to a cone plus a truncated cone and *D. acuminata* to a flattened ellipsoid. Biovolume estimates were based on the equations used in Olenina et al. [98]. Biovolumes of the two *M. rubrum* strains and *T. amphioxeia* were estimated with a particle counter (Multisizer 3 Coulter counter, Beckman, Roissy Charles De Gaulle, France) using triplicates of 10 mL for each species.

### 4.4. Experiment 1 (Baseline Study)

A preliminary experiment was carried out at IEO-Vigo to gain baseline information on the growth curves and metabolomes of the organisms. Triplicates of *Dinophysis acuminata*, *D. acuta*, *M. rubrum*, and *T. amphioxeia* where grown in 250 mL flasks filled with diluted (1:20) L1-Si medium [99] based on autoclaved seawater from Ría de Vigo with the salinity adjusted to 32 and a 12:12 h L:D cycle. Cultures were provided a light intensity of ~250 µE m^−2^ s^−1^ and were kept in a temperature-controlled chamber at 15 °C. *Mesodinium rubrum* was fed *T. amphioxeia* at a 1:1 ratio (*Teleaulax:Mesodinium*) and *Dinophysis acuminata* and *D. acuta* were fed the Spanish *M. rubrum* at a 10:1 ratio (*Mesodinium:Dinophysis*). More prey was added to *Mesodinium* when no cryptophytes were observed under the light microscope. To carry out toxin and non-targeted analyses, cultures that were harvested at mid-exponential phase were centrifuged at 3000× *g* for 20 min at 4 °C, the supernatant removed and the pellets kept at −80 °C before being sent on dry ice to IFREMER-Nantes.

### 4.5. Experiment 2 (Growth Curves)

Experiment 2 was carried out at IFREMER-Nantes and was developed in two phases, the first one lasting 7 days and the second 15 days, under identical experimental conditions. For the first phase (7 days), *Dinophysis* species, the two strains of *M. rubrum* and *T. amphioxeia* were seeded in triplicate into seawater from the English Channel off St. Malo, enriched with diluted (1:20) L1-Si medium in 250 mL Erlenmeyer flasks (150 mL final volume), and kept in a culture room at 17 °C with a 14:10 h L:D cycle at 160 μE m^−2^s^−1^. The two strains of *M. rubrum* were provided separately as prey for both *Dinophysis* species with a ratio 10:1 (*M. rubrum:Dinophysis*) on day 1. These were labeled as “well fed” treatments. The second phase (15 days) was carried out as previously described. The only difference was the longer duration of the second experiment (15 days), and that *Dinophysis* was fed only on day 0, and was therefore considered “prey-limited” at day 15 (starvation would have required being kept for weeks with no prey).

To estimate cell densities, samples of 10 mL (for Coulter counter) and 2 mL (sedimentation chambers) were taken every 2 days and were fixed with acidic Lugol’s solution. *Dinophysis* cells were counted in sedimentation chambers under a Zeiss Invertoskop D microscope (Carl Zeiss AG, Oberkochen, Germany), *Mesodinium* with a Coulter counter, and *T. amphioxeia*, with a Nageotte hemocytometer counting chamber.

Specific growth rates (µ, day^−1^) of *Dinophysis* species were calculated as:

µ = ln (*N_t_*/*N_o_*)/*t*

where *N_o_* was the initial and *N_t_* the final density of *Dinophysis* after time *t*.

### 4.6. Toxin Analysis

All the toxin analyses were performed at the Phycotoxins Laboratory (IFREMER-Nantes).

#### 4.6.1. Extraction and Hydrolysis

Samples from the first phase of Experiment 2 were collected during the exponential growth phase of each species, i.e., *T. amphioxeia* on day 3 and the *M. rubrum* controls and *Dinophysis* species on day 7, in Falcon tubes of 15 or 50 mL and centrifuged (Sigma 3-18K, Fisher Bioblock Scientific, France), as in Experiment 1. Pellets from Experiment 1 and 2 were extracted twice, resuspended in 0.5 mL MeOH, vortexed, and sonicated at 45 KHz for 15 min. Then, 200 mg of 200 μm glass beads were added and the mixture placed in a Mixer Mill MM 400 (Retsch GmbH, Haan, Germany) for 30 min to disrupt the cells, centrifuged at 12,000× *g* and 4 °C for 10 min and the supernatant (500 µL) filtered using 1.5 mL Eppendorf tubes with a 0.2 μm mesh included.For the second part of Experiment 2, extractions of each *Dinophysis* species triplicates that were fed each *M. rubrum* were carried out. Cultures of *M. rubrum* strains and the cryptophyte on day 12 were already senescent and discarded. For quantification, a basic hydrolysis of the *Dinophysis* and *M. rubrum* pellets from both experiments was carried out to convert esters of the main toxin (OA, DTX, and PTX) into their parent toxins (free toxins) [100]). For this purpose, 250 µL of methanol extracts of the samples were mixed with 30 µL of NaOH in hermetic-closing opaque-glass tubes and heated at 75 °C for 40 min. Samples were then left to cool down, their volumes verified and 30 µL of HCl added before being stirred, and then filtered through a 0.2 µm microfilter into 1.5 mL Eppendorf tubes, centrifuged (12,000× *g*, 10 min, 4 °C), and transferred to vials before injection.

#### 4.6.2. High Resolution Mass Spectral Analysis—System A: (QTOF 6550)

UPLC-HRMS analyses were carried out with a UHPLC system (1290 Infinity II, Agilent technologies, Santa Clara, CA, USA) coupled to a high resolution time-of-flight mass spectrometer (Q-Tof 6550 iFunnel, Agilent technologies, CA, USA), equipped with a Dual Jet Stream^®^ electrospray ionization (ESI) interface operating in both negative and positive mode in separate runs. Chromatographic separation was carried out on a reversed-phase C_18_Kinetex column (100 Å, 2.6 μm, 50 × 2.1 mm, Phenomenex, LePecq, France) at 40 °C using a mobile phase that was composed of water (A) and 95% acetonitrile/water (B) both containing 5 mM ammonium formate and 50 mM formic acid. The flow rate was set at 0.4 mL min^−1^ and the injection volume 3 µL. Separation was achieved using the following mobile phase gradient: from 10 to 50% B in 2 min, to 90% B over the next 3 min, held for 5 min before return to the initial condition (10% B) in 0.5 min, and a re-equilibration period (10% B) for 5.0 min. 

Mass spectral detection was carried out in full scan and targeted MS/MS mode in negative (ESI^−^) and positive (ESI^+^) ion acquisition. The full scan acquisition operated at a mass resolution of 40,000 Full Width at Half Maximum (FWHM) over a mass-to-charge ratio (*m/z*) range from 100 to 1700 with a scan rate of 2 spectra s^−1^. The targeted MS/MS mode was performed in a Collision Induced Dissociation (CID) cell using a mass resolving power of 40,000 FWHM over the scan range *m/z* from 50 to 1700 with a MS scan rate of 10 spectra s^−1^ and a MS/MS scan rate of 3 spectra s^−1^. Three different collision energies (i.e., 30, 50, and 70 eV) were applied to the precursor ions to obtain adequate fragmentation.

The conditions of the ESI source were set as follows: source temperature, 200 °C; drying gas, N_2_; flow rate, 11 mL min^−1^; sheath gas temperature, 350 °C; sheath gas flow rate, 11 mL min^−1^; nebulizer, 45 psig; capillary voltage, 3.5 kV; nozzle voltage, 500 V. A calibration-check was carried out continuously over the entire run time using reference masses *m/z* 121.0509 (purine) and *m/z* 922.0099 (hexakis phosphazine).

Acquisition (see Table 4) was controlled by Mass Hunter software (Agilent Technologies, CA, USA). Raw data were processed using the Molecular Feature Extraction (MFE) algorithm of the Agilent Mass Hunter Qualitative Analysis software (version B.07.00, service pack 1).

#### 4.6.3. Low Resolution Tandem Mass Spectrometry: System B (API 4000 QTrap)

UHPLC-LRMS/MS analyses were carried out with a UHPLC system (UFLC XR Nexera, Shimadzu, Tokyo, Japan) that was coupled to a hybrid triple quadrupole/ion-trap mass spectrometer (API 4000 QTrap, SCIEX, Redwood City, CA, USA), equipped with a turboV^®^ ESI source.

Toxins were separated using the same chromatographic conditions, as described above (system A). The injection volume was set at 5 µL. For quantitation, the mass spectrometer was operated in multiple reactions monitoring (MRM) acquisition mode, scanning two transitions for each toxin. Positive and negative acquisition experiments were established using the following source settings: curtain gas set at 30 psi (ESI^+^) and 20 psi (ESI^−^), ion spray at 5500 V (ESI^+^), and −4500 V (ESI^−^), Turbogas temperature of 550 °C, gas 1 and 2 set, respectively, at 40 and 50 psi, and an entrance potential of 10 V. Pectenotoxins (PTX) were analyzed in positive mode and OA group toxins in negative ionization mode (Table 4). Data acquisition and quantification were carried out using Analyst software version 1.6.2 (SCIEX, Redwood City, CA, USA). ANOVA was carried out at the end of the experiments to evaluate the statistical significance of toxin content per cell in Experiment 2.

Certified calibration solutions were purchased from the National Research Council Canada (NRCC, Halifax, NS, Canada): PTX2, OA, DTX1, and -2. HPLC grade methanol, acetonitrile, and formic acid (98%) were obtained from Sigma Aldrich (Steinheim, Germany). Ammonium formate was acquired from Fluka (St. Louis, MO, USA). Milli-Q water was obtained in-house to 18 MΩ cm^−1^ quality, using a Milli-Q integral 3 system (Merck Millipore, Guyancourt, France). For HRMS, acetonitrile and high purity water were obtained from Fisher Scientific (Illkirch, France).

### 4.7. Data Treatment for Non-Targeted Liquid Chromatography High Resolution Mass Spectrometry

#### 4.7.1. Feature Identification and Variables’ Selection

Features are distinct ion clusters, belonging either to compounds or to provisionally identified metabolites, which possess an abundance, a mass, a retention time, and, if present in available databases, a score thatallows their qualitative identification and assignment of a sum formula. Features were extracted from raw data files by a molecular feature extraction (MFE) algorithm using a Mass Hunter Workstation Qualitative Analysis Software (Agilent Technologies, Santa Clara, CA, USA) to create a work-flow to subtract procedural blanks in batches of samples (ESI^+^ and ESI^−^ ionization modes in extracted and hydrolyzed samples). An abundance cut-off value was selected so that features with peak heights of 5000 or higher were transformed into compound exchange format (.cef) files with the *Mass Hunter Reprocessor*, and were subsequently analyzed with *Mass Profiler Professional* software (MPP, version B 13.1.1) to identify statistically meaningful differences of features between groups. Compound alignment parameters were: RT = 0.0% + 0.15 min and mass = 15.0 ppm + 2.0 mDa.

Resulting abundance data were normalized using external scalars of each triplicate sample group. After this normalization, organisms were treated as equal-sized organisms and size effects on the results were avoided (e.g., compounds abundance on the smallest organism—in this study, *Teleaulax amphioxeia*—would be too small to allow for a comparison with the largest organism, *Dinophysis acuta* in this case. External scalars were calculated, as follows:

B = Biovolume X/Biovolume *Teleaulax* (1)

A = N_tot_ × (1) (2)

S = (2)/N_tot*Teleaulax*_ (3)

where B is the biovolume of species X normalized in relation to that of *Teleaulax*, A is the normalized biovolume of the total number of cells in the sample (N_tot_), S is the external scalar that was used for abundance data extracted from LC-HRMS.

Compounds satisfying a fold change cut-off of 2.0 and a 100% of frequency in each sample group triplicate were used to identify common and unique features within or between groups.

Sample quality assurance was tested by Principal Component Analysis (PCA). Significance testing was carried out using one-way and two-way ANOVA, an asymptotic *p*-value computation, a multiple testing correction of Benjamini-Hochberg, and a *p*-value cut-off of 0.001.

Similar and specific compounds were selected manually and verified with the *Find Similar Entities* tool displaying expression profiles of entities whose correlation coefficients to the target profiles were above the correlation cut-off range (0.5–0.85, 1.0) using an Euclidean similarity measure. *P*-values for “in both positive and negative ESI modes” were calculated with MPP, according to the variables “origin” (Spain and Denmark) and “feeding-status” (well-fed and prey-limited) for all the features in the samples. Samples list and grouping following the foregoing variables are shown in Table S12. Features were grouped into one variable depending on their distribution pattern. For features that were fulfilling the same conditions as above, only *p*-values of <0.001 were used.

Resulting compounds were identified with the *Agilent Mass Hunter ID Browser B.07.00* against databases (METLIN and the Dictionary of Marine Natural Products) with a mass-match tolerance of 2.00 ppm, and formulae were generated with the *Molecular Formula Generator* (MFG). Compound identification default settings were as follows: mass and retention time (RT) score were 100.00; isotope abundance and spacing scores were 80.00. Selected expected data variations were: 1.0 mDa + 2.0 ppm for MS mass, MS isotope abundance of 7.5%, MS/MS mass of 5.0 mDa + 7.5 ppm, and a RT of 0.115 min. For positive ions, neutral water losses and H^+^, Na^+^; K^+^, and NH_4_^+^ adducts were selected as charge carriers, whereas for negative ions, neutral water losses, and H^−^, Cl^−^, Br^−^, HCOO^−^, CH3COO^−^, and CF3COO^−^ were selected as charge carriers.

Compounds were clustered once they were identified following (a) “physiological meaning”: common and specific compounds of all and each organism, respectively, and (b) “compounds first found in other marine/terrestrial organisms” that were identified either in both or particularly in one of the *Dinophysis* species.

## 5. Conclusions

Metabolomic profiling allowed for us to differentiate *Dinophysis* species and treatments (“prey origin” and “nutritional status”, especially in *D. acuminata*), and some of the compounds that were involved were tentatively identified. Non-targeted analyses serve as a powerful screening tool and led to the identification of a new diol ester in *D. acuta*. The variable “species” gave the highest separation between groups of *D. acuminata* and *D. acuta*, followed by the variable “prey origin” (Danish and Spanish *M. rubrum* strain). Future studies should help in building up a “*Dinophysis*-metabolites library” initiated by this study, to confirm or to reject some of the hypotheses that are formulated in this paper. This library would provide valuable information regarding (i) metabolic fingerprints and pathways; (ii) predator-prey-recognition mechanisms related to target metabolites; and, (iii) clarify biogeographical distributions as well as the biological origin of compounds often identified first in higher, filter-feeding marine organisms, such as sponges.

## Figures and Tables

**Table 1:** Composition and collection day of the pellets obtained from Experiment 2.

Sample	Day	Cells (N) ± Std Dev.	Pellet Biomass % Average			Volume (mL)
			*Dinophysis*	*M. rubrum*	*T. amphioxeia*	
*T. amphioxeia*	3	283,333 ± 28,867			100	10
Danish *M. rubrum*	7	89,500 ± 866		100		15
Spanish *M. rubrum*	7	80,000 ± 8660		100		30
*D. acuta* + Danish *M. rubrum*	7	40,140 ± 1247	93.48	6.52		90
*D. acuta* + Spanish *M. rubrum*	7	29,943 ± 3264	97.26	2.74		130
*D. acuminata* + Danish *M. rubrum*	7	115,470 ± 9490	99.40	0.60		90
*D. acuminata* + Spanish *M. rubrum*	7	58,413 ± 3134	86.62	10.38		130
*D. acuta* + Danish *M. rubrum*	15	21,967 ± 2627	81.62	18.38		100
*D. acuta* + Spanish *M. rubrum*	15	42,733 ± 3225	100			100
*D. acuminata* + Danish *M. rubrum*	15	225,800 ± 9616	100			100
*D. acuminata* + Spanish *M. rubrum*	15	58,900 ± 265	100			100

**Table 2:** Toxin cell contents per biovolume and per cell (average ± standard deviation, *n* = 3) of *D. acuminata* and *D. acuta* cells from the baseline experiment (Experiment 1, mid-exponential growth phase) and from cultures under different nutritional status and prey (Experiment 2). Abbreviations: Sp = Species, PO = Prey origin, GP = Growth Phase, NS = Nutritional Status, ME = mid-exponential, ES = Spain, DK = Denmark.

Sp	PO	GP/NS	Toxin Contents pb = per Biomass (fg µm^−3^), pc = per Cell (pg cell^−1^) ± Standard Deviation									
			Free OA		Total OA		Free DTX2		Total DTX2		PTX2	
			pb	pc	pb	pc	pb	pc	pb	pc	pb	pc
*D. acuta*	ES	ME	0.17 ± 0.03	9.2 ± 2.0	0.22 ± 0.03	12.2 ± 2.3	0.07 ± 0.01	3.8 ± 0.6	0.08 ± 0.01	4.4 ± 0.9	0.41 ± 0.14	22.2 ± 9.4
*D. acuminata*	ES	ME	1.05 ± 0.34	21.5 ± 0.5	2.1 ± 0.3	35.2 ± 6.8						
*D. acuta*	ES	Well-Fed	0.55 ± 0.06	30.0 ± 3.7	0.75 ± 0.07	41.0 ± 4.9	0.24 ± 0.03	13.5 ± 1.7	0.32 ± 0.06	17.4 ± 4.1	0.69 ± 0.12	38.0 ± 8.2
		Prey-limited	1.49 ± 0.24	76.7 ± 18.6	1.36 ± 0.11	74.1 ± 8.2	0.56 ± 0.11	30.2 ± 8.5	0.60 ± 0.05	32.4 ± 3.8	1.29 ± 0.15	59.3 ± 11.8
	DK	Well-Fed	0.53 ± 0.12	28.7 ± 8.1	0.66 ± 0.11	35.9 ± 7.07	0.26 ± 0.03	14.2 ± 2.2	0.30 ± 0.01	16.5 ± 0.8	0.78 ± 0.01	70.0 ± 0.8
		Prey-limited	0.64 ± 0.08	34.6 ± 5.4	0.71 ± 0.07	38.6 ± 4.5	0.32 ± 0.04	17.4 ± 2.7	0.35 ± 0.03	19.0 ± 2.3	0.8 ± 0.09	43.6 ± 6.2
*D. acuminata*	ES	Well-Fed	0.37 ± 0.14	6.0 ± 3.0	0.36 ± 0.13	6.0 ± 2.8						
		Prey-limited	1.02 ± 0.20	17.1 ± 4.6	1.28 ± 0.03	21.5 ± 0.7						
	DK	Well-Fed	0.49 ± 0.09	8.3 ± 1.5	0.59 ± 0.08	9.8 ± 1.7						
		Prey-limited	1.44 ± 0.32	24.0 ± 6.5	1.93 ± 0.23	32.3 ± 4.7						

**Table 3:** Sequences of primers used for the genetic analysis of *Mesodinium.*

Name	Sequence 5′–3′	Reference
4617F	TCCTGCCAGTAGTCATATGC	[93]
Meso580R	GACGTACAGACTACGGACG	[94]
Meso245F	CGACTCGACGTCCCG	[94]
UNIDEUK1416R	GTTTCAGACTTGTGTCCATACTA	[95]
Meso580F	CGTCCGTAGTCTGTACGTC	[94]
Meso1480R	CTAAACACTCGATCGGTAGG	[94]
Meso1200F	ATTCCGGTAACGAACGAGAC	[94]
Meso28S_R	AGACTTGGATGACTTTTATCACC	[94]
ITS1	TCCGTAGGTGAACCTGCGG	[96]
Dir-2CR	CCTTGGTCCGTGTTTCAAGA	[97]

**Table 4:** Optimized parameters for the liquid-chromatography coupled to low resolution mass spectrometry (LC-LRMS/MS). MS acquisition parameters for API4000QTrap (System B). * fragment ion used for quantitation.

Toxin	Ionization Mode	Ion	Parent Ion (*m*/*z*)	Fragment Ions (*m*/*z*)	DP (V)	CE (eV)
OA	ESI^−^	[M − H]^−^	803.4	255.1 *	−170	−62
				113.1		−92
DTX2	ESI^−^	[M − H]^−^	803.4	255.1 *	−170	−62
				113.1		−92
DTX1	ESI^−^	[M − H]^−^	817.5	255.1 *	−170	−68
				113.1		−92
PTX2	ESI^+^	[M + NH_4_]^+^	876.6	823.6 *	91	31
				805.6		37
				213.6		55
PTX2sa and 7-epi PTX2sa	ESI^+^	[M + NH_4_]^+^	894.6	823.6 *	91	31
				805.6		37
				213.6		55
PTX1, PTX4 and PTX11	ESI^+^	[M + NH_4_]^+^	892.6	839.6 *	91	31
				821.4		37
				213.6		55
PTX3	ESI^+^	[M + NH_4_]^+^	890.5	873.6 *	91	31
				856.6		37
				213.6		55
PTX6 and PTX7	ESI^+^	[M + NH_4_]^+^	906.6	871.6 *	91	31
				853.6		37
				213.6		55
PTX12 and PTX14	ESI^+^	[M + NH_4_]^+^	874.6	857.6 *	91	31
				840.6		37
				213.6		55

## Supplementary Materials

The following are available online at http://www.mdpi.com/1660-3397/16/5/143/s1. Compounds highlighted in blue color correspond to compounds with an annotation immediately on the right of the row in which they are placed. Table S1A: Compounds common to all species in Experiment 1; Table S1B: Compounds common to all species in Experiment 2; Table S2: *T. amphioxeia* compounds from Experiment 2 (ESI^−^); Table S3A: Compounds occurring in both species of *Dinophysis* but specific to “prey origin” (ESI^+^), fold-change > 2, *p* < 0.001. Table S3B: Compounds differing between the two species of *Dinophysis* with fold-change > 2, *p* < 0.001 (ESI^+^), Table S3C: Compounds common to both species of *Dinophysis*, differentially expressed (fold-change > 2, *p* < 0.001) with interaction between “prey origin” and “species” (ESI^+^); Table S4A: Compounds common to both species of *Dinophysis*, differentially expressed (fold-change > 2, *p* < 0.001) as a function of “prey origin” (ESI^−^); Table S4B: Compounds common to both species of *Dinophysis*, differentially expressed (fold-change > 2, *p* < 0.001) as a function of “species” (ESI^−^); Table S4C: Compounds common to both species of *Dinophysis* differentially expressed (fold-change > 2, *p* < 0.001) with interactions between “prey origin” and “species” (ESI^−^); Table S5A: Statistically significant compounds common to both species of *Dinophysis* presented as a function of “species”, “prey origin” and “nutritional status” in ESI^−^; Table S5B: Statistically significant compounds common to both species of *Dinophysis* presented as a function of “species”, “prey origin” and “nutritional status” in ESI^+^; Table S6A: Compounds specific to in *Dinophysis acuta* (fold-change > 2, *p* < 0.001) according to “prey origin” and “nutritional status” in ESI^−^, Table S6B: Compounds specific to *Dinophysis acuta* (fold-change > 2, *p* < 0.001) as a function of “prey origin” and “nutritional status” in ESI^+^, Table S7A: Compounds specific to *Dinophysis acuminata* (i.e., differentially expressed with a fold-change > 2 and *p* < 0.001) according to “prey origin” and “nutritional status” in ESI^−^; Table S7B: Compounds found only (*p* < 0.001) in *Dinophysis acuminata* according to “prey origin” and “nutritional status” in ESI^+^; Table S8: The only statistically significant compound found in *Dinophysis acuta* in well-fed conditions (Danish *M. rubrum*)” in ESI^−^; Table S9A: Statistically significant compounds found in *Dinophysis acuta* in prey-limited conditions (Danish *M. rubrum*)” in ESI^−^; Table S9B: Compounds specific to *Dinophysis acuta* (fold-change > 2, *p* < 0.001) in prey-limited conditions (Danish *M. rubrum*)” in ESI^+^; Table S10: Compounds specific to *Dinophysis acuminata* (fold-change > 2, *p* < 0.001) in well-fed conditions (Danish *M. rubrum*)” in ESI^−^; Table S11A: Compounds specific to *Dinophysis acuminata* (fold-change > 2, *p* < 0.001) in prey-limited conditions (Danish *M. rubrum*)” in ESI^−^; Table S11B: Compounds specific to *Dinophysis acuminata* (fold-change > 2, *p* < 0.001) in prey-limited conditions (Danish *M. rubrum*)” in ESI^+^; Table S12: Samples resulting from Experiments 1 and 2 clustered according variables “Species”, “Prey origin” and “Nutritional Status”. PB: Procedural Blank; QC: Quality control; n/a: non-applicable; TA: *Teleaulax amphioxeia*; MR: *Mesodinium rubrum*; DATA: *Dinophysis acuminata*; DA: *Dinophysis acuta.*
